# Dietary Transforming Growth Factor-Beta 2 (TGF-β2) Supplementation Reduces Methotrexate-Induced Intestinal Mucosal Injury in a Rat

**DOI:** 10.1371/journal.pone.0045221

**Published:** 2012-09-12

**Authors:** Shani Ben-Lulu, Yulia Pollak, Jorge Mogilner, Jacob Bejar, Arnold G. Coran, Igor Sukhotnik

**Affiliations:** 1 Laboratory of Intestinal Adaptation and Recovery, The Bruce Rappaport Faculty of Medicine, Technion-Israel Institute of Technology, Haifa, Israel; 2 Department of Pediatric Surgery, Bnai Zion Medical Center, Haifa, Israel; 3 Department of Pathology, Bnai Zion Medical Center, Haifa, Israel; 4 Section of Pediatric Surgery, C.S. Mott Children's Hospital and University of Michigan Medical School, Ann Arbor, Michigan, United States of America; National Institutes of Health, United States Of America

## Abstract

**Background/Aims:**

Dietary supplementation with transforming growth factor-beta (TGF-β) has been proven to minimize intestinal damage and facilitate regeneration after mucosal injury. In the present study, we evaluated the effects of oral TGF-β2 supplementation on intestinal structural changes, enterocyte proliferation and apoptosis following methotrexate (MTX)-induced intestinal damage in a rat and in a cell culture model.

**Methods:**

Caco-2 cells were treated with MTX and were incubated with increasing concentrations of TGF-β2. Cell apoptosis was assessed using FACS analysis by annexin staining and cell viability was monitored using Trypan Blue assay. Male rats were divided into four experimental groups: Control rats, CONTR- TGF-β rats were treated with diet enriched with TGF-β2, MTX rats were treated with a single dose of methotrexate, and MTX- TGF-β rats were treated with diet enriched with TGF-β2. Intestinal mucosal damage, mucosal structural changes, enterocyte proliferation and enterocyte apoptosis were determined at sacrifice. Real Time PCR and Western blot were used to determine bax and bcl-2 mRNA, p-ERK, β-catenin, IL-1B and bax protein expression.

**Results:**

Treatment of MTX-pretreated Caco-2 cells with TGF-B2 resulted in increased cell viability and decreased cell apoptosis. Treatment of MTX-rats with TGF-β2 resulted in a significant increase in bowel and mucosal weight, DNA and protein content, villus-height (ileum), crypt-depth (jejunum), decreased intestinal-injury score, decreased level of apoptosis and increased cell proliferation in jejunum and ileum compared to the untreated MTX group. MTX-TGF-β2 rats demonstrated a lower bax mRNA and protein levels as well as increased bcl-2 mRNA levels in jejunum and ileum compared to MTX group. Treatment with TGF-β2 also led to increased pERK, IL-1B and β-catenin protein levels in intestinal mucosa.

**Conclusions:**

Treatment with TGF-β2 prevents mucosal-injury, enhances p-ERK and β-catenin induced enterocyte proliferation, inhibits enterocyte apoptosis and improves intestinal recovery following MTX-induced intestinal-mucositis in rats.

## Introduction

Mucositis is the term used to describe the damage caused to mucous membranes of the alimentary tract by radiation and chemotherapy, in particular with drugs affecting DNA synthesis (such as fluorouracil, methotrexate, and cytarabine) [1], [2]. The epithelium in the small intestine is extremely sensitive to cytostatic drug treatment, since it is proliferating rapidly. The loss of intestinal epithelial integrity causes pain and ulceration, vomiting, bloating, diarrhoea, symptoms of malabsorption, and an enhanced risk of bacteremia. The clinical presentations depend on the area of the alimentary tract affected. Mucositis limits the patient's ability to tolerate chemotherapy or radiation therapy, prolongs hospital stay, increases re-admission rates, compromises the patient's nutritional status, affects the patient's quality of life, and is occasionally fatal. Although treatment is available for a small subset of patients suffering from mucositis, the majority rely on pain relief as their only treatment option [3].

Over the last decade, significant progress has been made in understanding the pathophysiology underlying the development of mucositis. The current hypothesis for the development of mucositis was described by Keefe et al in 2004 [4], [5] and includes five biological phases, namely: 1) initiation, occurring following administration of cytotoxic chemotherapy; it encompass the primary damage response and is a result of DNA and non-DNA damage and the generation of reactive oxygen species (ROS); 2) message generation, involving the up-regulation of transcription factors including NFκB and subsequent activation of cytokine and stress response genes; 3) signaling and amplification, producing proteins, such as tumour necrosis factor (TNF), interleukin-1β (IL-1β) and interleukin-6 (IL-6), which cause direct tissue damage and provide positive feedback to amplify the process; 4) ulceration, resulting in painful ulcers, bacterial infiltration and an influx of macrophages and other inflammatory cells; and 5) finally healing, which spontaneously occurs upon cessation of chemotherapy.

In the intestinal mucosa, numerous cytokines have been shown to affect epithelial cell differentiation and proliferation through epithelial-mesenchymal and epithelial-immune cell interaction. The mammalian transforming growth factor family consists of three closely related members, designated TGF-β1, β-2 and β-3, all of which are potent inhibitors of epithelial cell growth [6], [7]. The TGF-beta family appears to play key regulatory functions in a diverse spectrum of biological processes, including modulation of proliferative activity of virtually all mammalian cell populations, cellular differentiation, embryological development of many tissues, and formation of extracellular matrix. The dietary formula containing TGF-β (Modulen) has been proven to have significant clinical utility in Crohn's patients by minimizing intestinal damage and facilitating regeneration after mucosal injury. Since severe inflammation of the intestinal mucosa plays a significant role in the development of chemotherapy-induced mucositis and is a major characteristic of the condition, we hypothesized in this study that dietary TGF-β2 supplementation would ameliorate methotrexate (MTX) induced intestinal mucosal damage when provided before MTX administration and would also stimulate intestinal recovery following MTX-induced mucositis in a rat and in a cell culture model.

## Materials and Methods

### Materials

Recombinant human TGF-β2 was purchased from Sigma-Aldrich, Israel. The factor has greater than 97% purity by SDSPAGE and HPLS analyses with endotoxin.

### Cell cultures

The human colorectal carcinoma cell line (CaCo-2) was grown to near confluence in 150 ml flasks in 5% CO_2_ at 37^°^C in Dulbecco's modified Eagle's medium supplemented with 10% fetal calf serum, 1% glutamine, 25 mM HEPES buffer, and 1% penicillin and streptomycin. Prior to the experiments, cells was trypsinised, washed and incubated in serum-free medium for 24 h. The serum-free medium was then replaced with that containing the experimental stimuli.

### Cell cycle analysis

The percentages of cells in the different phases of the cell cycle was determined by evaluating DNA content as was previously described [8]. To arrest cells at the G_1_/S border, cells were synchronized in a medium containing 2 mM hydroxyurea (Sigma) for 14 h. Cells were then transferred into fresh, hydroxyurea-free medium, or medium containing 0.4 µg/ml NBT-272. Control untreated or treated with TGF-α cells were harvested 0, 8, 16, and 24 h after release from hydroxyurea. After washing twice in PBS 19, the cells was stained with a solution containing 50 µg/ml of propidium iodide (PI) (Sigma-Aldrich, Israel) and 100 U/ml RNase A (Sigma-Aldrich, Israel) in PBS 19 for 30 min, at room temperature. A total of 30,000 events per sample were acquired. Flow cytometric analysis was performed on a FACS Caliber flow cytometer. The percentages of the cells in the different phases of the cell cycle were calculated on linear PI histograms using the mathematical software ModFit LT 2.0 (Verity Software House; Topsham, ME, USA).

### Cell apoptosis

For evaluation of cell apoptosis with TGF-β, cells were plated on six well cluster trays at a density of 10^4^cells/cm^2^ and examined 7 days after plating. Cells were pretreated with MTX for 24 h. Cultures pretreated with MTX and untreated cultures were then supplemented with TGF- β in concentrations ranging from 0.1 to 0.5 ng/ml, which was added to the luminal compartment for 24 h in medium. Apoptosis was assessed using FACS analysis at 24 h by annexin staining and at 48 h by propidium iodide (PI). For annexin staining, cells was trypsinised and re-suspended in 1 ml annexin-binding buffer (Pharmingen) and 5 µl Annexin V-FITC (2.5 µg/ml; Pharmingen) was added to the luminal compartment. After incubation in the dark at room temperature for 15 min, 50 µl PI (50 µg/ml; Sigma) was added to discriminate dead cells and the samples were analyzed on a FACS Caliber flow cytometer (Becton Dickinson). At least 12,000 cells were examined in the gated region and used for calculation. Dual parameter cytometric data was analyzed using CellQuest software from BD Biosciences (San Jose, Ca, USA). Viable cells are primarily Annexin V-FITC- and PI-negative; PI-positive staining indicates necrosis, Annexin V-FITC-positive staining indicates early apoptosis, and cells that are Annexin V-FITC- and PI-positive are considered to be in late apoptosis.

### Cell viability

The Caco2 cell lines were maintained in RPMI 1640 medium supplemented with 10% fetal calf serum, and were incubated in a humidified incubator at 37°C in 5% CO2. Experiments were initiated when the cells reached 80% confluence. Alamar Blue reduction test [9] was used for investigation of cell viability. Caco2 cells were seeded onto a 96-well plate with a density of 40×10^3^ cells/well and were further incubated under standard cultivation conditions (37°C, 95% air, 5% CO2). After an initial 24 h incubation to allow cellular attachment, cells were cultured in the medium with 0.5% fetal calf serum, and they were treated with 0.1 and 0.5 ng/ml TGF-β 2 for 48 h or with cell culture medium for 48 h (control and MTX). TGF-β 2 was dissolved in cell culture medium. After 48 h incubation, cells were cultured in the medium with 0.5% fetal calf serum, Pretreated with MTX 250 nm or non-treated cells were incubated with 0.1 and 0.5 ng/ml TGF-β 2 for 72 h or with cell culture medium for 72 h (control). After the treatment Alamar Blue solution was added directly in a final concentration of 10% and the plate was further incubated at 37°C for 3 h. Optical density of the plate was measured spectrophotometrically at a wavelength of 570 nm and 630 nm with a fluorescence reader (ELISA module, Anthos microplate spectrophotometer Zenyth 200, Anthos Labtec Instruments GmbH, Salzburg, Austria). Cell viability was calculated as percentage of the difference between the reductions of Alamar Blue in treated versus control cells. As a negative control, Alamar Blue was added to the medium without cells

### Animals

This experiment and animal care were conducted in compliance with the guidelines established by the “Guide for the Care and Use of Laboratory Animals”, Rappaport Faculty of Medicine, Technion (Haifa, Israel). Male Sprague-Dawley rats (250–300 g) were used in this study. Upon arrival, animals were housed in wire-bottomed cages and were acclimatized at 21°C on 12:12-h light-dark cycle for a minimum of five days before the experiment and were allowed access to water and chow ad libitum.

### Experimental design

Thirty two rats were divided randomly into four experimental groups of 8 rats each. 1) Group A– control rats underwent IP injection of 0.9% NaCl (CONTR); 2) Group B (CONTR-TGFβ) animals were treated with TGFβ2 supplemented chow 48 hours before and 72 hours after VEHICLE injection.; 3) Group C rats (MTX) were treated with a single IP injection of MTX (20 mg/kg); and 4) Group D (MTX- TGFβ) were treated with TGFβ2 supplemented chow similar to group B after the administration of MTX as in Group C. To determine the time of most severe intestinal damage, a single dose of MTX was injected and animals were sacrificed at day 1,3 and 7. Time-response curve has shown that day 1 represents the early phase of the induced intestinal damage, day 3 the phase of severe intestinal damage, and day 7 represents the regenerative phase. The day 3 was chosen in the studies described here.

### Intestinal mucosal parameters

All animals were sacrificed 72 hours following MTX or vehicle injection. The entire small intestine was carefully removed and placed on ice. Portions of intestine immediately distal to the ligament of Treitz and proximal to the ileo-cecal region were removed. Each segment was rinsed thoroughly with physiological saline and opened longitudinally on its antimesenteric border to expose the intestinal mucosa. The mucosa was collected and either processed immediately for histological analysis or snap frozen in liquid nitrogen for storage at −80°C for subsequent protein isolation. Total RNA, DNA and protein from the jejunum and ileum was extracted sequentially with a TRIzol reagent as previously described [10].

### Microscopic evaluation

Histological sections were prepared from the proximal jejunum (1 cm distal to the ligament of Treitz) and distal ileum (1 cm proximal to ileo-cecal junction) in all animals. Segments of small bowel were fixed for 24 h in 10% formalin, washed with absolute alcohol, and then processed into standard paraffin blocks. Five-micron transverse sections were prepared in a standard fashion and were stained with hematoxylin-eosin. The sections were studied microscopically using a micrometer eyepiece by an observer blinded as to the tissue's origin. Ten villi and crypts in each section were measured and the mean reading was recorded in microns, using a 10×4 magnifying lens. Histological images were loaded on a 760×570 pixels resolution buffer using a computerized image analysis system composed of a trichip RGB video-camera (Sony, Japan), installed on a light microscope (Zeiss, Germany) and attached to an IBM-compatible personal computer (Pentium III, MMX, 450 MHz, 125 MB RAM), equipped with a frame grabber. Images was captured, digitized, and displayed on a high-resolution color 17-inch monitor. The villus height and crypt depth were measured using the Image Pro plus 4 image analysis software (Media Cybernetics, Baltimore, MD). The degree of intestinal tissue injury was evaluated on a grading scale from 0 to 8 as described previously by Park et al [11]: 0-normal mucosa, 1-subepithelial space at villus tip, 2-more extended subepithelial space, 3-epithelial lifting along villus sides, 4-denuded villi, 5-loss of villus tissue, 6-rypt layer infarction, 7-transmucosal infarction, 8-transmural infarction.

### Enterocyte apoptosis

Immunohistochemistry for Caspase-3 was performed for identification of apoptotic cells using a combination of streptovidin-biotin-peroxidase method and microwave antigen retrieval on formalin-fixed, paraffin-embedded tissues according to manufacturers' protocols. Briefly, the sections were deparaffinized, rehydrated in graded alcohol, and microwave-pretreated in EDTA buffer for 10 min. Then the specimens were incubated in peroxidase quenching solution (3% H_2_O_2_ in methanol) for 10 min and blocked with serum blocking solution for 10 min. Thereafter, samples were stained with primary Caspase-3 cleaved polyclonal antibodies (diluted 1∶100, Biocare Medical, Walnut Greek, CA) for 60 min in a moist chamber at room temperature. After washing off the primary antibody in PBS, slides were incubated with a secondary human-absorbed, biotinylated, affinity-purified antibody. Enhanced horseradish peroxidase conjugated streptavidin was subsequently applied at room temperature for 10 min before the sections were visualized with DAB to create an intense brown deposit around the antigen–antibody–enzyme complex in the sample. The apoptotic index (AI) was defined as the number of apoptotic cells per 10 villi. Assessment was performed in a blinded manner by a qualified pathologist.

### Assessment of enterocyte proliferation

Crypt cell proliferation was assessed using 5-bromodeoxyuridine (5-BrdU). Standard BrdU labeling reagent (Zymed Laboratories, San Francisco, CA) was injected intraperitoneally at a dose of 1 ml/100 g body weight 2 h before sacrifice. Tissue sections were deparaffinized with xylene, rehydrated in graded ethanol solutions, and treated with 3% H_2_O_2_ methanol for 10 min at room temperature to remove endogenous peroxidase activity. Antigen unmasking was carried out by heating the sections for 10 min in 0.01 M sodium citrate (pH 6.0) at 100°C. Nonspecific protein binding was blocked using a buffer blocking solution. All sections were incubated using the primary mouse monoclonal antibodies anti-BrdU and the rabbit polyclonal secondary antibodies. Enhanced horseradish peroxidase conjugated streptavidin was subsequently applied at room temperature for 10 min before the sections were visualized with DAB to create an intense brown deposit around the antigen–antibody–enzyme complex in the sample. An index of proliferation was determined as the ratio of crypt cells staining positively for BrdU per 10 crypts.

### Immunohistochemistry for TGF-β receptor expression along the crypt-villus axis

Rat sections were fixed in fresh 4% paraformaldehyde for 4 h at 4°C. After deparaffinization, sections were treated with freshly prepared 0.6% H_2_O_2_ in methanol for 30 min to quench endogenous peroxidase activity. Sections were then treated with a biotinylated goat anti-rabbit antibody for 45 min and a biotin-avidin-peroxidase reagent for 30 min (Vectastain Elite kit; Vector Laboratories, Burlingame, CA). The primary antibody is an affinity purified rabbit anti-human type II TGF-β receptor polyclonal antibody which is nonreactive with the 53 kDa type I receptor (sc-400, Santa Cruz Biotechnology, Santa Cruz, CA). Incubations with rabbit IgG, as well as primary antibody preincubated for 15 min with synthetic type II TGF-β blocking peptide (10∶1 molar ratio) (supplied by Santa Cruz Biotechnology) were used as controls. Color was developed with diaminobenzidine (DAB) and sections were counterstained with hematoxylin.

### Western blotting

Tissue was homogenized in RIPA lysis buffer containing 50 mM Tris–HCl (pH 7.4), 150 mM NaCl, 1% NP-40, 2 mM EDTA, supplemented with a cocktail of protease and phosphatase inhibitors. Protein concentrations were determined by Bradford reagent according to the manufacturer's instructions. Samples containing equal amounts of total protein (15 µg) were resolved by SDS-PAGE under reducing conditions. After electrophoresis, proteins were transferred to a PVDF membrane and probed with various primary antibody to anti-bcl-2 antibody (1∶1000 dilution, sc-7382), anti-bax antibody (1∶200 dilution, sc-493), anti-phospho-ERK antibody (1∶2500 dilution, sc-7383), anti-β-catenin antibody (1∶1000 dilution, sc-7199), anti-TGF-β2 receptor antibody (1∶1000 dilution, sc-400), anti-ERK2 antibody (1∶1000 dilution, sc-56899), anti-IL-1B (1∶500 dilution) and anti-β-actin antibody (1∶1000 dilution)which were purchased from Santa Cruz Biotechnology (Santa Cruz Biotechnology Inc, CA). Horseradish peroxidase-conjugated secondary antibody was purchased from Jackson ImmunoResearch Laboratories Inc (West Grove, PA) and an enhanced chemiluminescent substrate from Biological Industries (Kibbutz Beth HaEmek, Israel). The optical density of the specific protein bands was quantified by using a densitometer (Vilber Lourmat, Lion, France).

### Expression of bax and bcl-2 genes (Real-Time PCR)

Expression of bax and bcl-2 levels was determined by quantitative real-time PCR (7500 Real-Time PCR System, Applied Biosystems, USA) on cDNA samples using Cyber Green Master Mix (ROVALAB, Germany) with the exception of template and primers. Primers for Rattus norvegicus bax and bcl-2 were synthesized by Syntezza Bioscience ltd. Israel, and 18 s rRNA Control kit was purchased from Eurogentec, EGT Group.

### Statistical analysis

The data are expressed as the mean ± SEM. A one-way ANOVA for comparison, followed by Tukey's test for pair-wise comparison was used for statistical analysis. Prism software was used (GraphPad Software, Inc., San Diego,CA) and statistical significance was defined as P<0.05.

## Results

### Cell apoptosis

**Figure 1:**
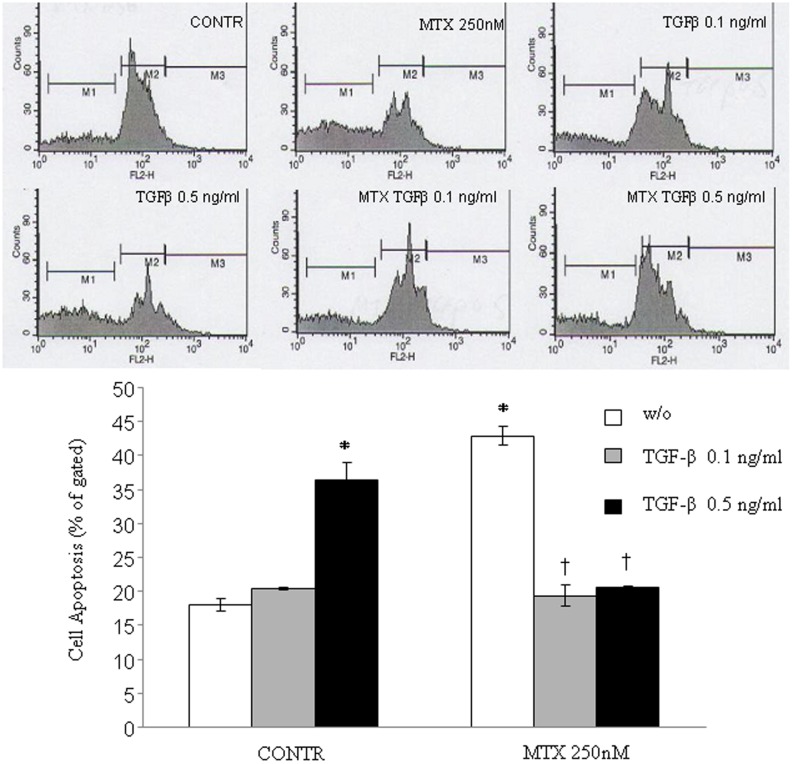
Effect of TGF-β on MTX-induced apoptosis in Caco-2 cells. Treatment of Caco-2 cells with MTX resulted in a significant increase in cell apoptosis compared to non-treated cells. Incubation of MTX-pre-treated Caco-2 cells with TGF-β (0.1 and 0.5 ng/ml) resulted in two-fold decrease in MTX induced cell apoptosis. Values are mean ± SEM. CONTR- control, MTX-methotrexate, TGF-β-transforming growth factor beta. *P<0.05 versus non-treated cells, ^†^P<0.05 MTX-TGF-β 0.1 ng/ml and MTX- TGF-β 0.5 ng/ml versus MTX pretreated cells.

Caco-2 cells were evaluated for apoptosis induction by PI staining. Incubation with TGF-β2 at concentration of 0.5 ng/ml resulted in a significant increase in apoptosis of CaCo-2 cells compared with medium only (1.5-fold increase in apoptosis, P<0.05) (Figure 1). Treatment with MTX resulted in a marked increase in cell apoptosis rates over corresponding control cells with vehicle alone (two-fold increase, P<0.05). Treatment of MTX-pretreated cells with TGF-β2 at concentrations of 0.1 ng/ml or 0.5 ng/ml resulted in a significant decrease in the apoptotic rate (two-fold decrease, P<0.05) compared with MTX-treated cells.

### Cell viability

**Figure 2:**
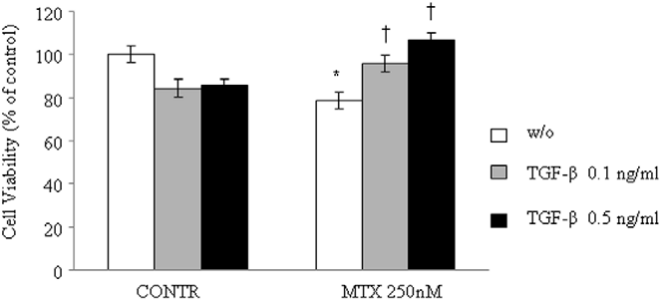
Effect of TGF-β and MTX on cell viability of Caco-2 cells (Alamar Blue reduction test). Cells were cultured in 96-well plates at a density of 4×10^4^ cells per well up to asubconfluent monolayer (80%), and then they were treated with 0.1 and 0.5 ng/ml TGF-β or control medium for 48 h,and then treated with 0.1 and 0.5 ng/ml TGF-β or MTX 125 nM, or 0.1 ng/ml and 0.5 ng/ml TGF-β with MTX 125 nM or control medium for 72 h. After the treatment, 20% of Alamar Blue was added to each well, and cells were incubated at 37°C for 3 h. Optical density was measured spectrophotometrically at 570 and 630 nm. Cell viability was calculated as percentage of the difference between the reductions of Alamar Blue in treated versus control. Results are presented as percentage of controls, mean ± SEM. CONTR-control, MTX- methotrexate, TGF-β- transforming growth factor beta. *P<0.05 MTX versus control rats, ^†^P<0.05 MTX-TGF-β 0.1 ng/ml and MTX-TGF-β 0.5 ng/ml versus MTX rats.

The changes in cell viability following exposure to MTX and TGF-β are shown in Figure 2. As expected, incubation of Caco-2 cells with 0.1 and 0.5 ng/ml concentrations of TGF-β resulted in non-significant decreased cell viability. Exposure of Caco-2 cells to MTX, 250 nm/ml, for 72 h resulted in a significant decrease in cell viability compared to control cells (21%, P<0.05). Exposure of MTX-pretreated cells to increasing concentrations (0.1 ng/ml or 0.5 ng/ml) of TGF-β for 48 and 72 hours led to an increase in cell viability (16% for 0.1 ng/ml TGF-β, P<0.05 and 27% for 0.5 ng/ml TGF-β, P<0.05) versus MTX-pretreated cells.

### Animal model

#### Body weight changes and stool patterns

**Figure 3:**
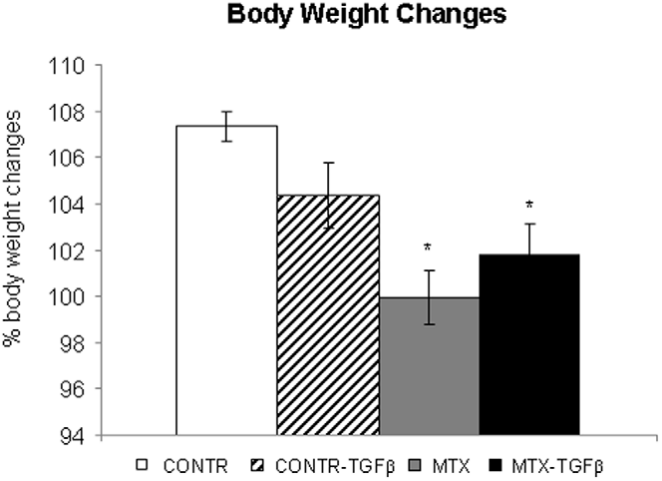
Effect of MTX and TGF-β2 on body weight change. Values are mean ± SEM. CONTR- control, MTX- methotrexate, TGF-β - transforming growth factor beta *P<0.05 MTX and MTX-TGF-β versus control rats.

Treatment with TGF-β2 (Group B) did not change significantly body weight compared to control animals (Group A) (Figure 3). MTX rats (Group C) demonstrated a significant decrease in final body weight (99±1 vs 102±0.9% initial, P<0.05) compared to control animals. Although MTX-TGF-β rats (Group D) demonstrated a trend toward an increase in final body weight compared to MTX animals (Group C), this trend did not achieve statistical significance. Eighty percent of MTX rats showed mild to moderate diarrhea. Treatment with TGF-β2 did not change stool patterns compared to MTX-non treated animals.

#### Intestinal mucosal parameters

**Figure 4:**
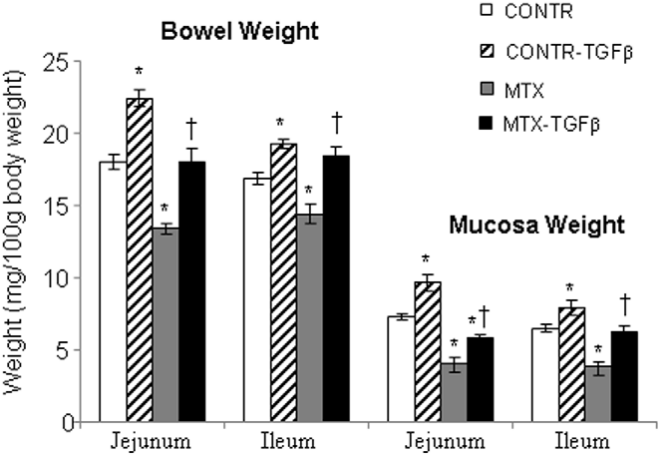
Effect of MTX and TGF-β on bowel and mucosal weight. Values are mean ± SEM. CONTR-control, MTX-methotrexate, TGF-β - transforming growth factor beta. *P<0.05 MTX and CONTR-TGF-β versus control rats. ^†^P<0.05 MTX-TGF-β versus MTX rats.

**Figure 5:**
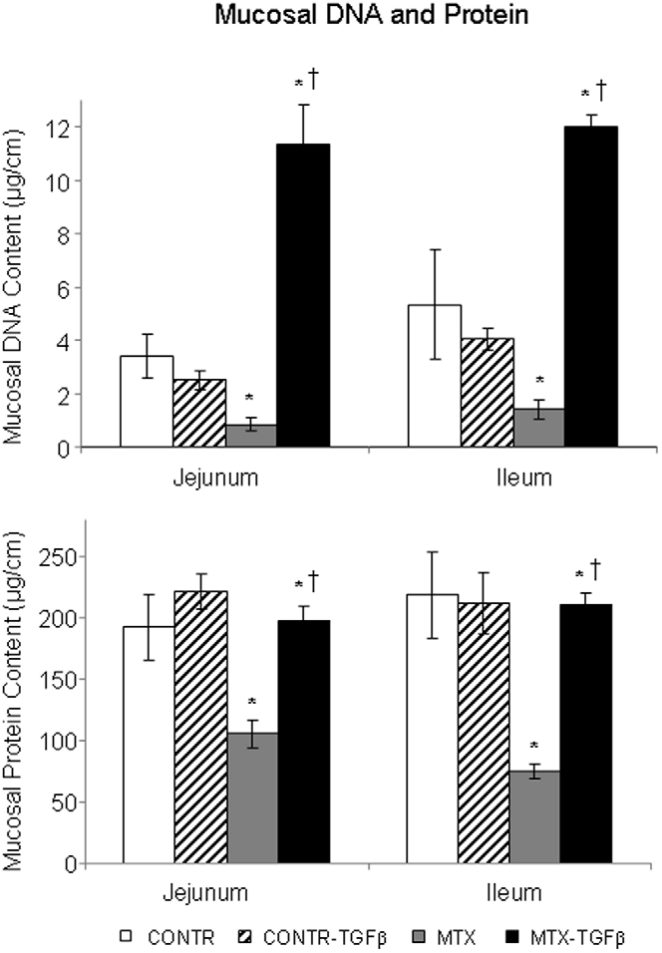
Effect of TGF-β on the mucosal DNA and protein content following MTX-induced mucositis. Values are mean±SEM. CONTR-control, MTX-methotrexate, TGF-β- transforming growth factor beta. *P<0.05 MTX and MTX-TGF-β versus control, ^†^P<0.05 MTX-TGF-β versus MTX rats.

Treatment with TGF-β2 (Group B) led to a significant increase in jejunal (20%, P<0.05) bowel weight, as well as in jejunal (25%, P<0.05) and ileal (18%, P<0.05) mucosal weight compared to control-untreated animals (Group A) (Figure 4). Treatment with MTX (Group C) resulted in a significant decrease in bowel weight in jejunum (26%, P<0.05) and ileum (15%, P<0.05), mucosal weight in jejunum (44%, P<0.05) and ileum (41%, P<0.05), mucosal DNA in jejunum (76%, P<0.05) and ileum (74%, P<0.05) (Figure 5), and mucosal protein in jejunum (46%, P<0.05) and ileum (66%, P<0.05) (Figure 5) compared to control animals (Group A). Treatment of MTX rats with TGF-β2 (Group D) led to a significant increase in jejunal (26%, P<0.05) and ileal (23%, P<0.05) bowel weight, jejunal (30%, P<0.05) and ileal (38%, P<0.05) mucosal weight, jejunal (93%, P<0.05) and ileal (89%, P<0.05) mucosal DNA content, and jejunal (47%, P<0.05) and ileal (65%, P<0.05) mucosal protein content when compared to MTX-animals (Group C).

### Intestinal histopathology

**Figure 6:**
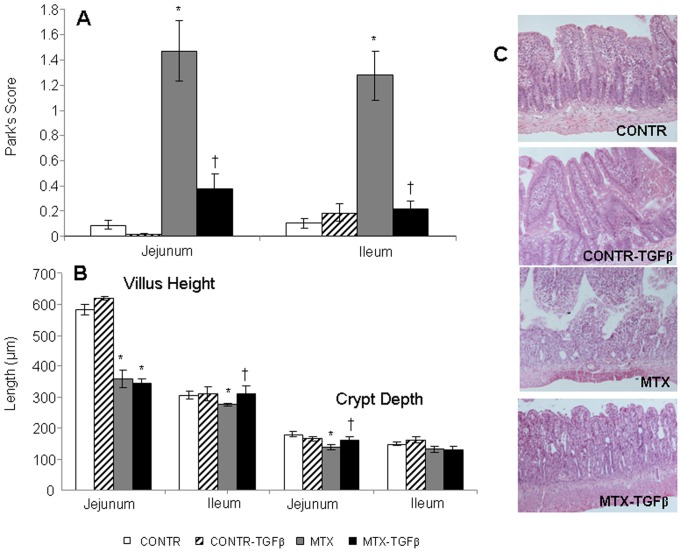
Effect of TGF-β on intestinal injury score and microscopic intestinal appearance following MTX-induced mucositis. The degree of intestinal tissue injury was evaluated on a grading scale from 0 to 8 as described previously by Park (A). The villus height and crypt depth were measured using the Image Pro plus 4 image analysis software (B). (**C**) Representative slides of MTX-induced intestinal injury. The histopathology analysis of the tissue sections from MTX-treated animals showed a significant epithelial atrophy, blunting of the villi and signs of crypt remodeling that was accompanied by marked cellularity mainly by mononuclear cells in the lamina propria, the presence of the flattened and vacuolated cells, and an increased number of blood vessels in the stroma. TGF-β2 administration resulted in less significant epithelial atrophy and crypt remodeling compared to MTX rats. Values are mean ± SEM. CONTR-control, MTX-methotrexate, TGF-β- transforming growth factor beta. *P<0.05 MTX and MTX-TGF-β versus control, ^†^P<0.05 MTX-TGF-β versus MTX rats. CONTR-control,

Treatment of control rats with TGF-β2 did not change significantly Park's score, villus height and crypt depth in jejunum and ileum compared to control non-treated animals (Figure 6). Microscopic analysis of the intestine 72 hours after MTX injection revealed a characteristic change of intestinal damage (Figure 6C), including a significant epithelial atrophy, blunting of the villi and signs of crypt remodeling which was accompanied by marked cellularity, mainly with mononuclear cells in the lamina propria, the presence of flattened and vacuolated cells, and an increased number of blood vessels in the stroma. Consistent with these findings, the intestinal injury score increased significantly in MTX rats in both jejunum (12-fold increase, P<0.05) and ileum (16-fold increase, P<0.05) compared to control rats (Figure 6A). Following TGF-β2 administration (Group D), MTX rats showed less significant inflammatory cell infiltration as well as less prominent epithelial atrophy and crypt remodeling. In accord, MTX- TGF-β rats manifested a significant decrease in the intestinal injury score in jejunum (6-fold decrease, P<0.05) and ileum (4-fold decrease, P<0.05) compared to MTX animals (Group C).

MTX-treated rats (Group C) demonstrated significantly shorter villus heights in jejunum (359±29 vs 584±18 µm, P<0.05) and ileum (277±4 vs 306±14 µm, P<0.05) as well as crypt depth in jejunum (138±10 vs 180±7 µm, P<0.05) compared to control rats (Group A) (Figure 6B). Treatment with TGF-β2 of MTX rats (Group D) was manifested by a significant increase in villus height in ileum (310±27 vs 277±4 µm, P<0.05) and crypt depth in jejunum (159±12 vs. 138±10 µm, P<0.05) compared to MTX animals (Group C).

### Cell proliferation

**Figure 7:**
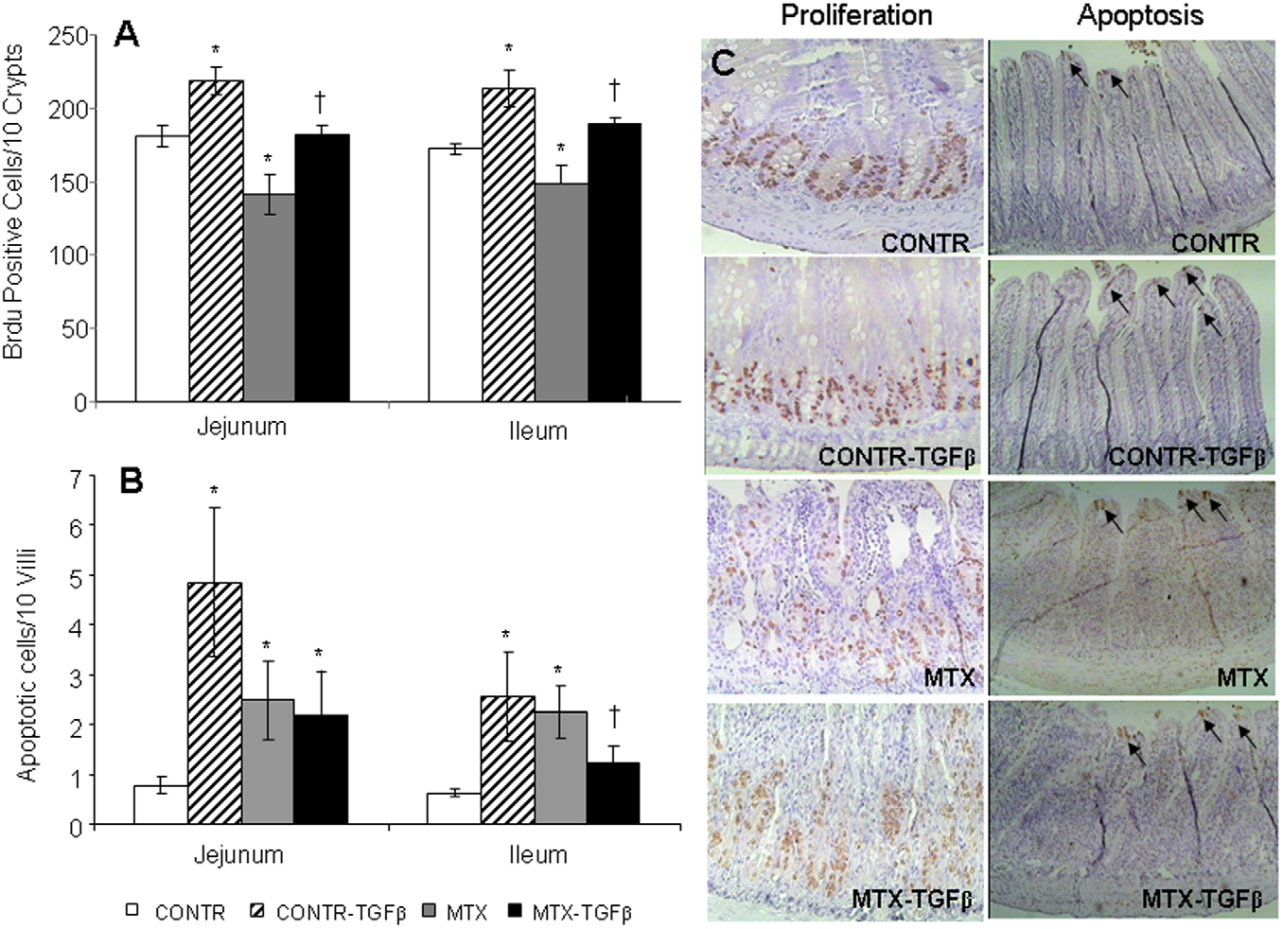
Effects of MTX and TGF-β on enterocyte proliferation (A) and apoptosis (B). Values are mean ± SEM. CONTR-control, MTX-methotrexate, TGF-β- transforming growth factor beta. *P<0.05 MTX and CONTR-TGF-β versus control, ^†^P<0.05 MTX-TGF-β versus MTX rats. (**C**) Representative slides of cell proliferation and apoptosis following TGF-β2 administration during MTX-induced mucositis. MTX-treated animals showed a significant decrease in cell proliferation rates and concomitant increase in cell apoptosis compared to control animals. TGF-β2 administration in MTX rats resulted in a significant increase in cell proliferation and decrease in cell apoptosis compared to MTX animals.

Figure 7 illustrates crypt cell proliferation (incorporation of 5-BrdU into DNA) in the four experimental groups. CONTR- TGF-β rats (Group B) demonstrated significantly greater cell proliferation rates in jejunum (219±9 vs 181±7 BrdU positive cells/10crypts, P<0.05) and ileum (213±12 vs 172±4 BrdU positive cells/10crypts, P<0.05) compared to control rats (Group A) (Figure 7A,C). Treatment with MTX resulted in a significant decrease in cell proliferation in both the jejunum (141±14 vs 181±7 BrdU positive cells/10crypts, P<0.05) and ileum (148±12 vs 172±4 BrdU positive cells/10crypts, P<0.05) compared to control animals. Following TGF-β2 administration (Group D), MTX animals demonstrated a significant increase in proliferation rate in the jejunum (183±6 vs 141±14 BrdU positive cells/10crypts, P<0.05) and ileum (190±4 vs 148±12 BrdU positive cells/10crypts, P<0.05) compared to the MTX group (Group C).

### Enterocytes apoptosis

Administration of TGF-β2 in control rats (Group B) resulted in a significant increase in cell apoptosis in jejunum (4.9±1.5 vs 0.8±0.2 apoptotic cells/10villi, P<0.05) and ileum (2.6±0.9 vs 0.6±0.1apoptotic cells/10villi, P<0.05) compared to control animals (Figure 7B,C). MTX-induced mucositis was accompanied by a significantly increased cell apoptosis in jejunum (2.5±0.8 vs 0.8±0.2 apoptotic cells/10villi, P<0.05) and ileum (2.3±0.5 vs 0.6±0.1apoptotic cells/10villi, P<0.05) compared to control animals. Treatment of MTX rats with TGF-β resulted in decreased cell apoptosis in ileum (1.2±0.3 vs 2.3±0.5 apoptotic cells/10villi, P<0.05) compared to MTX animals as well as in a trend toward a decrease in cell apoptosis in jejunum; however, this decrease was not statistically significant.

### Western blot for TGF- β receptor

**Figure 8:**
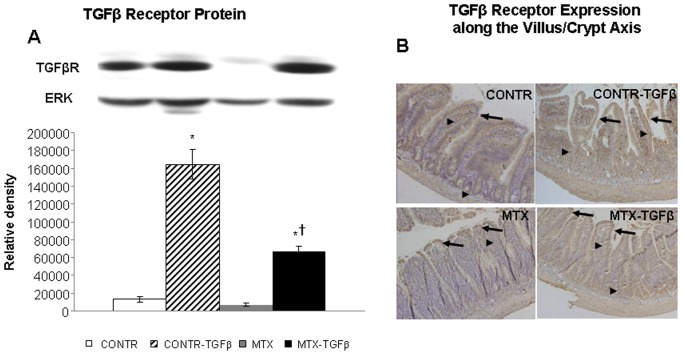
Effects of MTX and TGF-β on TGF-βR protein. Relative expression of TGF-βR protein in jejunum, as determined by Western blot analysis, of rats following MTX induced mucositis (A). Values are mean ± SEM. CONTR–control, MTX-methotrexate, TGF-β-transforming growth factor beta. *P<0.05 MTX-TGF-β and CONTR-TGF-β versus control rats, ^†^P<0.05 MTX-TGF-β versus MTX rats. ERK was detected using anti-ERK antibody to verify equal protein loading. (**B**) Immunohistochemistry showing the expression of the type II TGF-β receptor along the crypt-villus axis in jejunum. Sections were incubated with an affinity purified rabbit anti-rat type II TGF-β receptor polyclonal antibody which is non-reactive with the type I receptor. Color was developed as described in Materials and methods. There was no change in the expression of the receptor along the villus-crypt axis (arrows-expression in villus, arrowheads- expression in crypt) in control rats. Treatment with MTX resulted in a lower type II TGF-β2 receptor expression compared to control animals. Treatment of control and MTX animals with TGF-β2 resulted in a more significant receptor expression compared to control and MTX-non-treated animals, both in the crypt region and along the entire villus, especially at the basolateral side of enterocytes. The staining was more prominent in differentiated villus tip cells compared to crypt cells. The changes in distribution of Type II TGF-β2 receptor was correlated with the protein levels observed in the Western blot analysis.

CONTR-TGF-β rats (Group B) demonstrated a significant increase in Type II TGF-β receptor protein (10-fold increase, P<0.05) as compared to control animals (Group A) (Figure 8A). Treatment with MTX resulted in a trend toward a decrease in Type II TGF-β receptor protein as compared to control rats. Following TGF-β administration, MTX-TGF-β animals (Group D) demonstrated a significant increase in Type II TGF-β receptor protein (5-fold, P<0.05) as compared to MTX and control rats.

### TGF-β receptor type II expression along the villus-crypt axis

Type II TGF-β2 receptor along the villus-crypt axis was examined using immunohistochemistry (Figure 8B). Sections were incubated with an affinity purified rabbit anti-rat type II TGF-β2 receptor polyclonal antibody which is non-reactive with the type I receptor. In control rats, the lamina propria and the villous base area contained weak staining for TGF-β2 receptor and weak diffuse reaction against TGF-β2 could be detected within the enterocytes consistent with earlier observations (12). There was no change in the expression of the receptor along the villus-crypt axis. Treatment of control animals with TGF-β2 resulted in a more significant receptor expression compared to control-non-treated animals. The membranous staining in villus enterocytes was more prominent compared to the enterocytes within the crypts. Treatment with MTX (Group C) resulted in a lower type II TGF-β2 receptor expression compared to control animals. Similar to CONTR-TGF-β, MTX-TGF-β rats showed much more intense staining for the TGF-β2 receptor, both in the crypt region and along the entire villus, especially at the basolateral side of the enterocytes. The staining was more prominent in differentiated villus tip cells compared to crypt cells. The changes in distribution of Type II TGF-β2 receptor were correlated with the protein levels observed in Western blot analysis.

### Expression of Bcl-2 and Bax genes (Real Time PCR)

**Figure 9:**
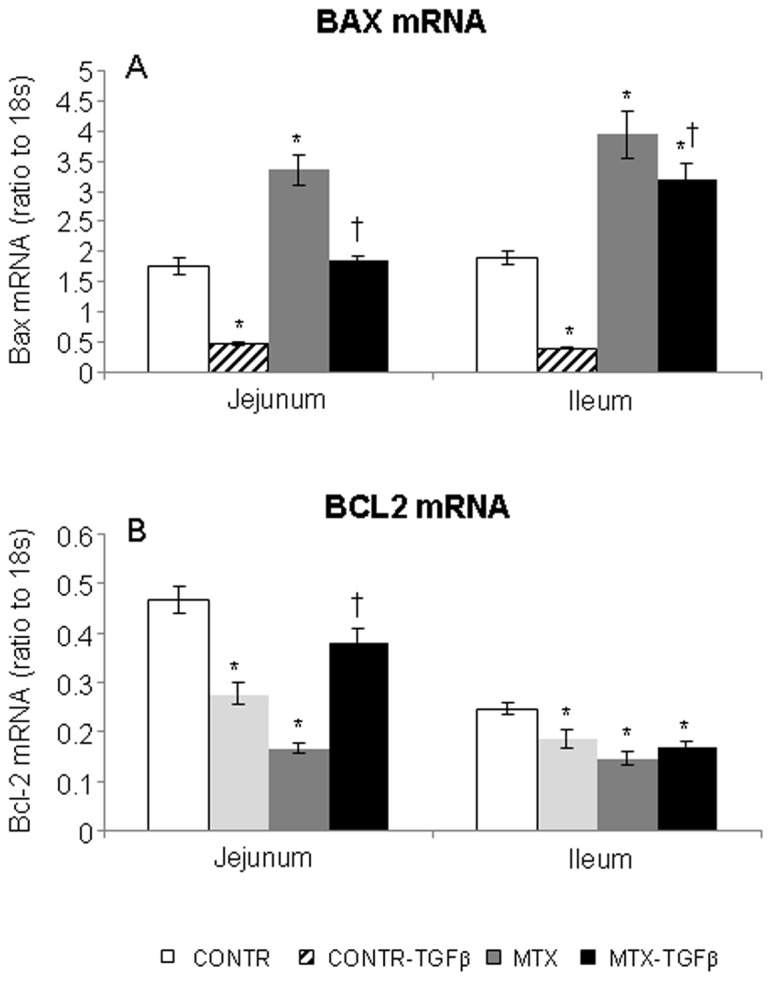
Effect of TGF-β on Bax mRNA (A) and Bcl-2 mRNA (B) expression in gut mucosa following MTX-induced intestinal mucositis. Results are expressed as the ratio of the investigated mRNA over 18S mRNA expression. Values are mean ± SEM. CONTR-control, MTX- methotrexate, TGF-β -transforming growth factor beta. *P<0.05 MTX, MTX-TGF-β and CONTR-TGF-β versus control rats, ^†^P<0.05 MTX- TGF-β versus MTX rats.

Control-TGF-β animals demonstrated a significant decrease in bax mRNA expression in jejunum (3-fold decrease, P<0.05) and ileum (3-fold decrease, P<0.05) as compared to control rats (Figure 9A). Following MTX administration, bax mRNA expression was up-regulated in jejunum (two-fold increase, P<0.05) and ileum (two-fold increase, P<0.05) compared to control animals. Treatment of MTX-rats with TGF-β2 attenuated the pro-apoptotic effects of MTX. MTX-TGF-β rats showed a significant decrease in bax mRNA expression in the jejunum (42%, P<0.05) and ileum (20%, P<0.05) compared to MTX-animals.

Treatment with MTX resulted in a significant down-regulation of bcl-2 mRNA levels in jejunum (2.5 fold decrease, P<0.05) compared to control rats (Figure 9B). MTX-TGF-β rats showed a significant increase in a bcl-2 mRNA expression in jejunum compared to MTX-animals (two-fold increase, P<0.05) as well as a trend tonward a increase in the bcl-2 mRNA expression in ileum; however this trend was not statistically significant.

### Western blot

**Figure 10:**
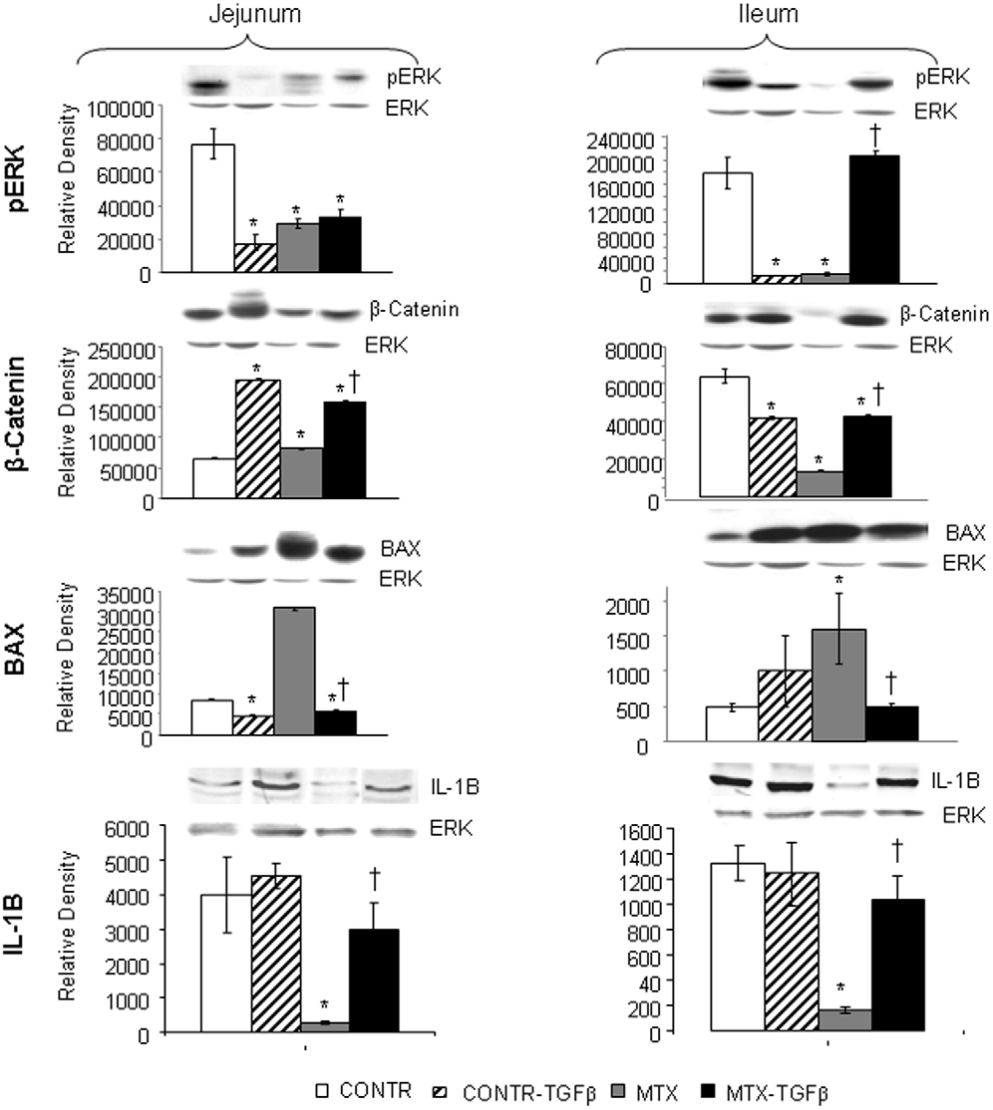
Effect of MTX and TGF-β on p-ERK, β-catenin, IL-1B and Bax protein levels. Relative expression of p-ERK, β-catenin, IL-1B and Bax proteins in jejunum and ileum as determined by Western blot analysis of rats following MTX induced mucositis. Values are mean ± SEM. CONTR-control, MTX-methotrexate, TGF-β -transforming growth factor beta. *P<0.05 MTX, MTX-TGF-β and CONTR-TGF-β versus CONTR rats, ^†^P<0.05 MTX- TGF-β versus MTX rats. ERK2 was detected using anti-ERK2 antibody to verify equal protein loading.

In jejunum, Western blot analysis (Figure 10) illustrated a significant decrease in p-ERK protein levels (marker of proliferation) in MTX compared to control animals. These findings correlate with decreased rates of cell proliferation in MTX animals compared to control rats. Interestingly, β-catenin protein levels increased in MTX rats compared to control animals. This finding may reflect the activated stem cell activity and may suggest the initiation of a regenerative process 72 hours after MTX-induced damage. Treatment with TGF-β resulted in a significant increase in β-catenin protein levels as compared to MTX and control rats. Bax protein levels (marker of apoptosis) increased significantly in MTX rats as compared to control rats and was correlated with elevated bax mRNA levels as well as with elevated rates of cell apoptosis following MTX administration. Treatment with TGF-β resulted in a significant decrease in bax protein levels compared to control and MTX rats. MTX animals have shown a significant decrease in IL-1B protein levels compared to control animals. Treatment with TGF-β resulted in a significant increase in IL-1B protein levels compared to MTX rats.

In ileum, Western blot analysis (Figure 10) illustrated a significant decrease in p-ERK protein, IL-1B protein and β-catenin protein levels in MTX rats compared to control animals. These findings correlated with decreased rates of cell proliferation in MTX animals compared to control rats. Treatment with TGF-β resulted in a significant increase in p-ERK, IL-1B and β-catenin protein levels compared to MTX animals. Similar to jejunum, bax protein levels increased significantly in ileum of MTX rats as compared to control rats. Treatment with TGF-β resulted in a significant decrease in bax protein levels compared to MTX rats.

## Discussion

The regulation of crypt cell proliferation, migration along the crypt-villus axis, entrocyte differentiation, and cell death via apoptosis are maintained through the complex interaction of many different factors including nutrients, hormones and peptide growth factors. Over the past decades, an increasing number of peptide growth factor genes have been identified, and some have been fully sequenced, cloned and synthesized. Several distinct peptide families are now known to modulate different cell functions of intestinal cell populations, including cell proliferation and differentiation [13]. The effect of growth factors in preventing intestinal damage by cytostatic drugs has not been well described and the mechanisms of this effect have not been clearly investigated. Very little is known about the absorptive and protective functions of epithelial cells during damage and regeneration induced by chemotherapy.

Intestinal cell turnover is normally regulated by reciprocal interactions between the epithelium and the underlying mesenchymal stroma. The formation of villi is normally preceded by the condensation of mesenchymal cells adjacent to the epithelium in crypts at the presumptive sites of new villus formation. Changes in the stromal environment may indirectly contribute to changes in the number and size of the crypts as well as allow their progressive invasion of villus tissue [14]–[16]. Many different signals contribute to those phenotypic changes that are associated with maturation of the gut epithelium. One such signal consists of an autocrine/paracrine regulatory loop that involves transforming growth factor-beta (TGF-β) [17], [18]. The major site of expression for TGF-β in the gastrointestinal tract is the epithelial layer and lamina propria of the small intestine and underlying mesenchymal stroma [19]. The mammalian TGF-β family consists of three closely related members, designated TGF β_1_, β_2_ and β_3_, all of which are potent inhibitors of epithelial cell growth [6], [7]. These cytokines have been implicated in diverse phenomena including growth control, cell adhesion and motility, production of extracellular matrix components, and alteration of cell phenotype [19]–[21]. TGF-β has been found to bind to several specific cell surface TGF-β receptors. TGF-β receptor is a serine/threonine kinase receptor complex that consists of two distinct transmembrane proteins known as type I and type II receptors [19], [22]. The type-1 and type-2 receptors work in a cooperative fashion; ligand binding to the type-1 receptor facilitates activation of the associated type-2 receptor, which then activates the intracellular signaling machine via SMAD proteins.

Harsha et al have recently demonstrated that the use of Modulen, an immunomodulatory dietary supplement containing TGF-β, glutamine, and short chain fatty acids, provides a protective role against MTX-induced mucositis in a rat model [23]. The authors showed that administration of formula supplemented with TGF-β provided statistically significant protection against weight loss, hypoalbuminemia, acidosis, and gastrointestinal damage following MTX administration. The authors hypothesized that Modulen supplementation resulted in one or more of the following: (1) the protection of epithelial stem cells against MTX damage, leading to a larger mucosal stem cell population postregimen, (2) increased regeneration during and after the regimen because of escape of mitotically-active cells from apoptosis, and (3) the acceleration of regeneration after cessation of the MTX treatment.

The purpose of the current study was to evaluate the effect of TGF-β2 on enterocyte turnover during MTX-induced intestinal mucositis in a rat and in cell culture model. The molecular mechanisms underlying the dynamic processes of intestine-specific gene expression, cell fate determination, cellular differentiation and intestinal development were evaluated. IN the Caco-2 cell culture, treatment with MTX resulted in a marked increase in cell apoptosis rates and a concomitant decrease in cell viability over corresponding control cells treated with vehicle alone. Although the main effect of MTX is an inhibition of cell proliferation, recent evidence suggests that MTX induces cell apoptosis in cell lines [24] and that this pro-apoptotic effect is correlated to an elevation in Bax/Bcl-2 ratio [25]. The effects of TGF-β2 on cell apoptosis were different in the non-treated and MTX-treated Caco-2 cells. Incubation of nontreated Caco-2 cells with TGF-β2 resulted in a significant decrease in viability and an increase in apoptosis in the CaCo-2 cells compared with medium only. The pro-apoptotic effect of TGF-β2 was dose-dependent with the maximal effect observed at a concentration of 0.5 ng/ml. The anti-proliferating and pro-apoptotic effects of TGF-β on epithelial cells have been described [26]. Biazi et al have demonstrated that CaCo-2 cells are sensitive to the growth-inhibitory effects of TGF-β, secondary to a marked enhancement of apoptosis through c-Jun N-terminal kinase and Smad4 activities [27].Treatment of MTX-pretreated cells with TGF-β 0.1 ng/ml or 0.5 ng/ml resulted in a significant two-fold decrease in the apoptotic rate and a concomitant increase in cell viability compared with MTX-pretreated cells. These changes were in agreement with previous findings that demonstrated the inhibitory effects of TGF-β2 on cell apoptosis in different cell types, including cerebellar granule cell precursors and osteoblasts [28], [29]. The mechanisms of the anti-apoptotic effect of TGF-β remain unclear. In a recent experiment, Singla et al have demonstrated that TGF-β2 treatment of mouse embryonic stem cells resulted in a two- to fivefold increase in cytoprotective released factors (interleukin-10, stem cell factor, tissue inhibitor of matrix metalloproteinase-1, and VEGF) and inhibit iodoacetic acid and H_2_O_2_-induced apoptosis in the cell culture system [30]. Recent evidence suggests that the FasL-Fas-caspase extrinsic apoptosis pathway is regulated by the TGF-β signaling cascade and is essential for organ development [31].

Since exposure to TGF-β2 inhibited cell apoptosis and enhanced cell viability, we next investigated the effect of TGF-β2 on cell turnover during MTX-induced intestinal mucositis in a rat model. Animals were injected with a single IP dose of MTX (20 mg/kg) and were treated with TGFβ2 supplemented chow 48 hours before and 72 hours after MTX injection. BrdU was used in our experiment to determine an index of crypt cell proliferation. This analogue of thymidine is incorporated into the DNA of proliferating cells during the S-phase of the cell cycle. Immunohistochemistry for caspase-3 was used to characterize enterocyte apoptosis.

Treatment of control animals with dietary TGFβ2 supplementation exerted a positive effect on the small intestinal mucosa. This is evident from increased overall bowel and mucosal weight in jejunum and ileum as well as from increased rates of cell proliferation. This finding is contrary to several reports of the inhibitory effects of TGFβ on epithelial cell proliferation in cell lines [7], [8]. It should be emphasized that the positive effect of TGFβ on interactions between the epithelium and the underlying mesenchymal stroma predominates over the direct inhibitory effects of TGFβ on epithelial cell proliferation. Proliferating cells are restricted to crypts that are deeply embedded in the submucosal mesenchyme. As cells begin to differentiate, they migrate towards the lumen and are eventually shed, either from the tips of the intestinal villi or from the surface of the intestinal epithelium. One can hypothesize that changes in the stromal environment following TGFβ2 administration may indirectly contribute to changes in the cell proliferation within the crypts and allow their progressive invasion of villus tissue. The mild stimulatory effect of TGFβ2 on cell proliferation in our study was accompanied by elevated β-catenin protein levels, which may suggest an activation of stem cell activity within the crypt following changes in the stromal environment. Our data demonstrated the elevated rates of cell apoptosis following TGFβ2 administration that, together with elevated cell proliferation, may represent accelerated cell turnover. We have also shown a significant decrease in anti-apoptotic bcl-2 gene expression which may be responsible for enhanced cell apoptosis which is correlated with our in vivo study and with data from the literature [25]. Interestingly, the expression of the pro-apoptotic gene bax was also down-regulated in TGF-β rats compared to control animals. We believe that the decreased bcl-2 expression drives increased cell apoptosis, while the down-regulation of bax mRNA creates a higher resistance of enterocytes to apoptosis. The other explanation of this phenomenon may be the different rates of apoptosis at multiple time points following development of mucositis. Consistent with this concept is the observation that in the first few days MTX may down-regulate the bcl-2 gene expression that drives elevated cell apoptosis [25]. After three days, the bulk of apoptotic enterocytes appear, leading to down-regulation of bax mRNA in attempt to decrease cell death, while the bcl-2 gene still remains decreased. These findings suggest an important role for the differential regulation of apoptosis related genes as coordinators of the early increase in cell apoptosis after MTX-induced damage.

Consistent with our previous experiments, MTX-induced mucositis in the current study resulted in apparent intestinal damage. This conclusion is supported by the observed increase in the Park injury score compared to control animals. MTX rats also showed severe villous atrophy, epithelial flattening, and extensive crypt loss. In addition, treatment with MTX resulted in significant mucosal hypoplasia. A decrease in bowel and mucosal weight, a decrease in mucosal DNA and protein, and decrease in villus height and crypt depth support this conclusion. Parallel decreases in mucosal DNA and protein indicate that the smaller mucosal mass of MTX animals can be attributed to cellular hypoplasia. Histologically, villus height and crypt depth decreased in response to MTX administration, suggesting decreased absorptive surface area. We also observed strong inhibitory effects of MTX on enterocyte proliferation, which may be considered as a major mechanism responsible for decreased intestinal cell mass and mucosal hypoplasia. As a folic acid analogue, the action of MTX primarily inhibits DNA synthesis by binding to the enzyme dihydrofolate reductase. This leads to an inhibition of proliferation in the crypts of the small intestine [32]. These changes were in agreement with previous findings that demonstrated marked damage in the crypt epithelium at days 1 and 2 after MTX administration, while days 3 and 4 represented a phase of prominent damage to the villous epithelium, marked by reduced cell and villous heights, and villous atrophy [33]. A decreased cell proliferation rate in MTX animals was accompanied by decreased levels of p-ERK protein levels. The transmission of extracellular proliferation and differentiation signals into their intracellular targets is mediated by a signaling cascade culminating in mitogen-activated protein kinase (MAPK). One of the MAPK signaling pathways triggered by cytokines or growth factors is the extracellular signal-related kinase (ERK) pathway. The relationship between MTX and MAPK pathway has been described previously in various experimental models and clinical trials [34]. Cell loss in the small intestine with MTX-induced mucositis is mainly regulated by programmed cell death. Small intestinal crypt cells rapidly undergo apoptosis in response to cytotoxic drug treatment, which results in gastrointestinal toxicity [35]. The bcl-2 family has been implicated in both positive and negative regulation of intestinal cell apoptosis. There are strong indications from our results and from previously published data [36] that intestinal epithelial cell apoptosis increases significant following MTX administration. Our results show that the intrinsic pathway, with its regulation by the bcl-2 family of proteins, was altered by MTX consistent with changes in cell apoptosis. The mRNA and protein levels of the pro-apoptotic bax increased, while those of the antiapoptotic bcl-2 gene decreased. Correspondingly, the bax/bcl-2 ratio increased in MTX- rats compared to control animals, suggesting decreased enterocyte survival. Although elevation of producing proteins, such as tumour necrosis factor (TNF), interleukin-1β (IL-1β) and interleukin-6 (IL-6) has been described during the third phase of intestinal mucositis (signaling and amplification) [4], [5], our data suggest that MTX-induced mucositis was accompanied by a decreased levels of IL-1B protein levels. Further experiments are required to determine the role of other cytokines in development of mucositis.

Results of the current study show that dietary TGFβ2 administration protects the intestinal mucosa from damage caused by MTX. While MTX rats showed severe villous atrophy, extensive crypt loss, and signs of crypt remodeling, TGFβ2-treated rats showed more preserved architecture as well as the presence of newly formed crypts and regeneration. 80% of rats showed a significant decrease in intestinal mucosal injury grade compared to MTX animals, suggesting lesser degrees of intestinal damage. In addition, exposure to enteral TGFβ2 accelerated intestinal mucosal repair and enhanced enterocyte turnover. While the proliferative zone in MTX-rats moved progressively upwards in the crypts toward the crypt-villus junction, the proliferative zone of MTX- TGFβ2 rats was only mildly affected, showing a slight shift upwards within the crypts. In addition, exposure to oral TGFβ2 significantly enhanced intestinal recovery following MTX-induced damage. This is evident from the significant increase in bowel and mucosal weight, increased DNA and protein content in ileum. Histologically, marked increases in villus height (in ileum) suggest increased absorptive surface area and closely correlate with increased cell mass. Similar to control rats, TGFβ2 supplementation resulted in a significant increase in mucosal cell proliferation in functioning intestine, but decreased significantly cell apoptosis rate, which may represent the main mechanism that maintains mucosal structure following MTX-induced damage. Enhanced cell proliferation in the current study was correlated with elevated β-catenin and p-ERK protein levels that may suggest an activated stem cell activity and stimulated MAPK signaling pathway. Our results show also that the intrinsic pathway, with its regulation by the bcl-2 family of proteins, was altered by TGFβ2 in accordance with changes in cell apoptosis: the mRNA and protein levels of the pro-apoptotic bax decreased, while those of the antiapoptotic bcl-2 mRNA levels increased. Correspondingly, bax/bcl-2 ratio decreased in MTX- TGFβ rats compared to MTX animals, suggesting increased enterocyte survival. Further investigation is needed to define the regulation of this special apoptotic state with respect to the Fas/Fasl-mediated extrinsic pathway. This positive effect was accompanied by decreased levels of IL-1B protein in intestinal mucosa, suggesting anti-inflammatory effect of TGF- β2.

Next, we investigated whether the effects of TGF-β2 on enterocyte proliferation and apoptosis were correlated with TGF-β2 receptor expression throughout the gastrointestinal tract (Western blot) and along the villus–crypt axis (Immunohistochemistry). We have shown that the pro-proliferative and anti-apoptotic effect of TGF-β2 on enterocyte turnover is correlated with elevated TGF-β2 receptor expression following TGF-β2 administration (CONTR-TGF-β vs CONTR and MTX-TGF-β vs MTX). In the crypt compartment, a significant increase in TGF-β2 receptor expression following TGF-β2 administration coincided with increased cell proliferation. In villus tips, MTX- TGF-β rats demonstrated higher receptor immunoreactivity compared to MTX animals. Since TGF-β exerts anti-apoptotic effects, this increase in TGF-β2 receptor expression coincides with decreased cell apoptosis in villus tips following TGF-β2 administration.

In conclusion, treatment with TGF-β2 increased cell viability and decreased of cell apoptosis in Caco-2 cell line. In a rat model of MTX-induced mucositis, dietary TGF-β2 supplementation reverse intestinal damage, causes anti-inflammatory effect and stimulates intestinal recovery. Enhanced cell proliferation (through Wnt/ β-catenin and MAPK signaling pathways) and inhibited programmed cell death (through up-regulation of bcl-2 and down-regulation of bax expression) may be responsible for this effect. The pro-proliferative and anti-apoptotic effects of TGF-β2 are correlated with TGF-β2 receptor expression along the villus-crypt axis. Dietary TGF-β2 may be clinically beneficial as an agent to prevent intestinal damage and stimulate intestinal recovery in patients with chemotherapy-induced mucositis.
